# Biomass-fuelled improved cookstove intervention to prevent household air pollution in Northwest Ethiopia: a cluster randomized controlled trial

**DOI:** 10.1186/s12199-020-00923-z

**Published:** 2021-01-04

**Authors:** Mesafint Molla Adane, Getu Degu Alene, Seid Tiku Mereta

**Affiliations:** 1 Department of Environmental Health, College of Medicine & Health Sciences, School of Public Health, Bahir Dar University, Bahir Dar, Ethiopia; 2 Department of Epidemiology and Biostatistics, College of Medicine & Health Sciences, School of Public Health, Bahir Dar University, Bahir Dar, Ethiopia; 3 Department of Environmental Health Sciences and Technology, Jimma University, Jimma, Ethiopia

**Keywords:** Biomass-fuelled, Cookstove, Household air pollution, Particulate matter

## Abstract

**Background:**

Household air pollution from biomass fuels burning in traditional cookstoves currently appeared as one of the most serious threats to public health with a recent burden estimate of 2.6 million premature deaths every year worldwide, ranking highest among environmental risk factors and one of the major risk factors of any type globally. Improved cookstove interventions have been widely practiced as potential solutions. However, studies on the effect of improved cookstove interventions are limited and heterogeneous which suggested the need for further research.

**Methods:**

A cluster randomized controlled trial study was conducted to assess the effect of biomass-fuelled improved cookstove intervention on the concentration of household air pollution compared with the continuation of an open burning traditional cookstove. A total of 36 clusters were randomly allocated to both arms at a 1:1 ratio, and improved cookstove intervention was delivered to all households allocated into the treatment arm. All households in the included clusters were biomass fuel users and relatively homogenous in terms of basic socio-demographic and cooking-related characteristics. Household air pollution was determined by measuring the concentration of indoor fine particulate, and the effect of the intervention was estimated using the Generalized Estimating Equation.

**Results:**

A total of 2031 household was enrolled in the study across 36 randomly selected clusters in both arms, among which data were obtained from a total of 1977 households for at least one follow-up visit which establishes the intention-to-treat population dataset for analysis. The improved cookstove intervention significantly reduces the concentration of household air pollution by about 343 μg/m^3^ (*Ḃ =* − 343, 95% CI − 350, − 336) compared to the traditional cookstove method. The overall reduction was found to be about 46% from the baseline value of 859 (95% CI 837–881) to 465 (95% CI 458–472) in the intervention arm compared to only about 5% reduction from 850 (95% CI 828–872) to 805 (95% CI 794–817) in the control arm.

**Conclusions:**

The biomass-fuelled improved cookstove intervention significantly reduces the concentration of household air pollution compared to the traditional method. This suggests that the implementation of these cookstove technologies may be necessary to achieve household air pollution exposure reductions.

**Trial registration:**

The trial project was retrospectively registered on August 2, 2018, at the clinical trials.gov registry database (https://clinicaltrials.gov/) with the NCT03612362 registration identifier number.

**Supplementary Information:**

The online version contains supplementary material available at 10.1186/s12199-020-00923-z.

## Background

Household energy use is essential to human beings for preparing food, room heating, and lighting as well as for numerous other purposes. However, depending on its quality, household energy use also has harmful consequences due to toxic emissions [1] such as particulate matter, carbon monoxide, polycyclic aromatic hydrocarbons, and volatile organic compounds that result in household air pollution (HAP) [2].

There is sufficient evidence linking HAP to various health impacts [3–5]. In particular, HAP exposure from biomass fuel burning in traditional cookstoves (TCS) currently appears as one of the most important threats to public health [6], and it becomes among the leading risk factors for global morbidity and mortality currently [7]. The latest Global Burden of Disease Study 2016 estimates 2.6 million household air pollution-associated deaths, with the burden tremendously occurred in low- and middle-income countries (LMICs) [8].

The linkage of HAP exposure to various health impacts arises largely from the incomplete combustion of solid biomass fuels in TCSs which are known to cause a range of health impacts when inhaled [9, 10]. Nevertheless, solid biomass fuel burning in TCSs is still practiced by an estimated 3 billion people worldwide [11], and it remains one of the major methods of cooking in most LMICs [12].

In Ethiopia, more than 95% of houses used biomass fuels as the primary household energy source [13, 14] in TCSs [15–19]. As a result, even though the World Health Organization (WHO) air quality guideline [20] is set at a health-protective level, previous studies on the magnitude of HAP exposure documented much higher HAP concentrations than the guideline values. For example, a recent study found a concentration of 926.34 μg/m^3^ of sampled air for indoor fine particulate matter less than 2.5 micrometers in diameter (PM_2.5_) in Ethiopian households [21]. Other earlier studies also reported the worst findings such as mean indoor PM_2.5_ concentration of 1357 μg/m^3^ [22] measured among traditional biomass-fuelled cookstoves using households in Ethiopia.

Concerning HAP prevention, it is essential to highlight that the concentration of HAP emitted from indoor biomass burning depends on several factors such as the type of stove, kitchen characteristics, fuel type, quantity of fuel, ventilation, and method of cooking that could influence the magnitude of HAP [23, 24]. In an effort to minimize HAP concentration associated with biomass fuel use, improved cookstove (ICS) interventions have been advocated and widely practiced as a potential solution in LMICs [25, 26]. However, biomass-fuelled ICS is controversial because of its properties of solid fuel combustion [27], and a recent systematic review revealed that previous trials examining the effect of biomass-fuelled ICSs on HAP concentration demonstrate a high statistical variability between estimates [28].

Also, despite the significant HAP reductions, the post-intervention levels are well above the WHO guideline [29]. For example, previous randomized controlled stove trials reported significant reductions for micro-environmental PM_2.5_ concentration reductions to 485, 320, and 119 in μg/m^3^ following local ICS interventions in Rwanda [30], Ghana [31], and India [26], respectively, which are all higher than the current air quality guideline value [20].

Moreover, previous systematic reviews reported that some biomass-fuelled ICS interventions are not delivering results that are even close to the levels needed [24], and data from both laboratory and field settings suggest many of the biomass-fuelled ICSs currently on the market have limited benefit in terms of HAP reduction [32]. For example, a more recent ICS trial found no evidence that an improved biomass-fuelled cookstove intervention reduced personal exposure to PM_2.5_ concentrations in μg/m^3^ among cooks and children [33]. The up-to-date, systematic reviews also concluded that studies on the effect of biomass-fuelled ICS interventions in LMICs are still limited, heterogeneous, and inconclusive [28, 29], which suggested the need for further research.

The magnitude of HAP [21, 22] and its association with child health outcomes [13, 34] have been reported in several observational studies in Ethiopia. However, despite some pilot trial efforts [35], no community-level biomass-fuelled ICS trial research had been conducted in Ethiopia as evidenced by the recent systematic reviews [36, 37]. Therefore, the effect of the biomass-fuelled *Mirt* ICS intervention on HAP reduction attracted considerable attention, as it is a recognized commercially distributed type of ICS in Ethiopia [38, 39], and investigation into its effect on HAP reduction had not been adequately dealt with in earlier community-level trial studies emphasizing the need for further investigation.

Thus, given the high burden of HAP and lack of trial studies in Ethiopia, we conducted this cluster randomized controlled trial study to assess the effect of biomass-fuelled ICS intervention on the concentration of HAP compared with the continuation of an open burning TCS method. Consequently, this manuscript reports on the effect of the ICS intervention in a low-income community of Northwest Ethiopia, which is considered representative of the majority of households in the country. The study was addressed an important gap in cookstove trial evidence by testing whether the Ethiopian biomass-fuelled ICS intervention can significantly reduce HAP concentration or not.

## Materials and methods of the study

### Study locations and context

Ethiopia is situated in the Northeastern part of Africa and occupies an area of 1.1 million square kilometers ranging from 4620 m above sea level to 148 m below sea level [40]. The country possesses three major topographic-induced climatic zones, the hot lowlands (*Kolla*) located 1500 below, the temperate (*Wayna Dega*) which range 1500–2400, and the cool temperate highlands (*Dega*) located above 2400 m above sea level [40, 41].

The average annual temperature is approximately 15–20 and 25–30 °C for highlands and lowlands, respectively [41]. This trial was conducted in a low-income rural community of the Mecha Health and Demographic Surveillance System (MHDSS) site in Northwest Ethiopia. MHDSS site is a field research center established in 2013 by Bahir Dar University to carry out and support postgraduate level studies in the region. It is located 525 km away from the capital city of Ethiopia, Addis Ababa, towards Northwest and 40 km far away from the capital city of Amhara Regional State, Bahir Dar. According to the official population profile report of MHDSS, the study area comprises 132 clusters/*Gots* with a total of 65,086 populations within 20631 houses at the end of 2016.

Our earlier research work, carried out in the current study area, have also shown that all households use biomass fuel as the primary household energy source and the households in the included clusters are relatively homogenous in terms of basic socio-demographic and cooking-related characteristics [13, 19], which made them ideal populations for rationale comparison of the study groups in the current trial study. Furthermore, the presence of extra indoor (95.8%) and outdoor (38.1%) burning events such as coffee ceremony, burning incense, local alcohol/*areqi* making, burning rubbish, and charcoal production were common observable facts in the study area [13]. About 63% of the households use a separate kitchen, and most (89.1%) houses are owned privately [19].

### Study design

As part of the wider stove trial project in Northwest Ethiopia (ClinicalTrials.gov Identifier: NCT03612362), a community-level cluster randomized controlled trial study with two arms of equal size was used to assess the effect of biomass-fuelled ICS intervention on the concentration of HAP compared with the continuation of an open burning TCS method. Cluster is a small village, termed as *Got* in Amharic (both national and local language), is the lowest administrative unit in Ethiopia, and is used as the smallest unit of enumeration areas by the Ethiopian national census authority. All eligible households in the selected clusters were enrolled as control or intervention for baseline and repeated follow-up visits approximately every 3 months for 1 year after receiving the intervention. The concentration of HAP at the individual household level was measured before the installation of *Mirt* ICS and, again in the same households, 4 times after the intervention households received the ICS intervention. The households with the TCS method were served as a control arm.

### Eligibility criteria

All clusters/*Gots* and households under the MHDSS site were eligible for participation in the cookstove trial, and all households who were exclusive users of TCS for *injera* baking were eligible for participation in the trial. Only households who did not have any enclosed cooking quarter (kitchen) arrangement were excluded.

### Sample size determination

To estimate the effect of ICS intervention on HAP over the follow-up period, the sample size was calculated based on previous publication [42] by considering a detectable difference of 30% in HAP concentration reduction by the ICS intervention to be worth pursuing, a two-sided alpha of 0.05, a power of 80%, and the common coefficient of variation (CoV) value of 0.7 for HAP measurement outcome in biomass fuel using households [42]. Accordingly, the estimated sample size (*n*) was found to be 171 households in each arm assuming individual randomization.

However, since this trial randomized the intervention over clusters instead of individual houses, the standard formulae for estimation of sample size might lead to an underpowered study which may be inconclusive. Thus, the calculated sample size assuming the individual randomization was inflated by a design effect (DE) value to reach the required level of statistical power under cluster randomization using the formula [43]:

$$ \mathrm{DE}=1+\kern0.5em \left[\left({\mathrm{CoV}}^2+1\right)\mathrm{m}'-1\right]\ \mathrm{ICC}. $$

Considering a CoV value of 0.2 for cluster size and an average number of eligible houses of 55 from the updated data of MHDSS and an intra-cluster correlation coefficient (ICC) value of 0.05 for HAP to cope with the unknown ICC was suggested by previous reviews of ICC values [44, 45]. Accordingly, the DE value became about 4, and the required sample size for the cluster randomized controlled trial (*n*_c_) became about 684 households per arm using the formula:

$$ {\mathrm{n}}_{\mathrm{c}}=\mathrm{n}\kern0.5em \left[1+\left(\left({\mathrm{CoV}}^2+1\right)\ \mathrm{m}'-1\right)\ \mathrm{ICC}\right]. $$

Then, with an additional 30% to account for any unpredictable events in the field due to equipment-related problems as well as for any lost to follow-up (LTF) events such as unexpected change of cooking behavior of the participant houses [42, 46], the required sample size becomes about 978 houses per arm. Finally, the number of clusters (*K*) required in each arm for unequal cluster sizes was determined using the formula [47]:

$$ \mathrm{K}=\mathrm{n}\kern0.5em \left[1+\left(\left({\mathrm{CoV}}^2+1\right)\mathrm{m}'-1\right)\ \left.\mathrm{ICC}\right]/\mathrm{m}'\right] $$

, which became about 18 clusters per arm, and this caused to increase the sample size to 990 houses per arm.

### Randomization and masking

Clusters were randomly allocated to intervention and control arms at a 1:1 ratio by an independent epidemiologist using a computer-generated randomization schedule. Intervention status was revealed after all baseline measurements had been completed as well as all study households recruited and assigned to their respective arm to ensure the allocation sequence was concealed from those assigning the arms. Also, participating households and data collectors were blinded to intervention status during study enrollment and baseline data collection. All eligible households within the clusters were included in the study to minimize the risk of selection bias; however, because of the typical feature of cluster design and nature of the intervention under study, blinding of the households receiving ICS intervention was not possible.

The major rationale for adopting a cluster randomized trial design was to prevent contamination [48] or unintentional spill-over of intervention effects from one treatment group to another of the trial if individual household randomization was used, as the concentration of HAP would inevitably be affected by the air pollution status from neighbor households. The other rationales were to increase administrative effectiveness and minimize costs [49].

### Sampling and recruitment of households

The cluster sampling method was used to select 36 clusters randomly (18 clusters per arm) among the total 132 clusters in the MHDSS site, and all eligible households were included within the selected cluster (complete enumeration). The list of clusters/*Gots* and households was established from the MHDSS record, the selected households were identified using the permanent MHDSS site house number, and the actual participant households were recruited at the household level by field workers during the baseline survey after ensuring whether the households met the eligibility criteria.

A screening questionnaire was used by field data collectors upon their first visit to each household to ensure that the household was appropriate and willing to participate. When the household met the eligibility criteria, the study was explained to the heads of the household, and they were asked whether the household would be willing to participate in the study and use ICS technology for at least 12 months. Then, when the heads of the household agreed to be involved in the study, the field staff administered a written consent form at that time, and the consent procedure was conducted in Amharic (both national and local language). To achieve adequate participant enrolment, we utilized local energy experts and health extension workers to oversee the overall efforts in recruiting eligible houses.

### Intervention

#### Trial descriptions and implementation

In general, there are about six primary types of biomass-fuelled improved cookstoves [50]. These are as follows:

1. Rocket (also known as side-fed) cookstoves: these are fuelled with wood sticks or biomass residues that are continuously fed through the side of the stove, typically resting on a grate so that ash and charcoal can settle below. Air enters by natural or forced-draft through the same opening as the fuel (examples: Grameen Greenway Smartstove, Envirofit G-3300)
2. Gasifier cookstoves: these are batch-or-continuously fed using processed fuel (examples: Awamu Troika, Mimi Moto, and Philips ACE 1)
3. Charcoal cookstoves: these are batch-operated and fuelled with charcoal or carbonized biomass (examples: Kenyan Ceramic Jiko, Envirofit CH-2200, and Burn Jikokoa)
4. Forced-draft/fan cookstoves: these cookstoves have air that is forced into the stove using a fan or a blower to enhance turbulence and promote cleaner combustion (example: BioLite HomeStove)
5. Batch-operated cookstoves: these stove types are operated on a single load of fuel at a time
6. Continuously fed cookstoves: these stoves require fuel to be loaded throughout the cooking process

**Fig. 1:**
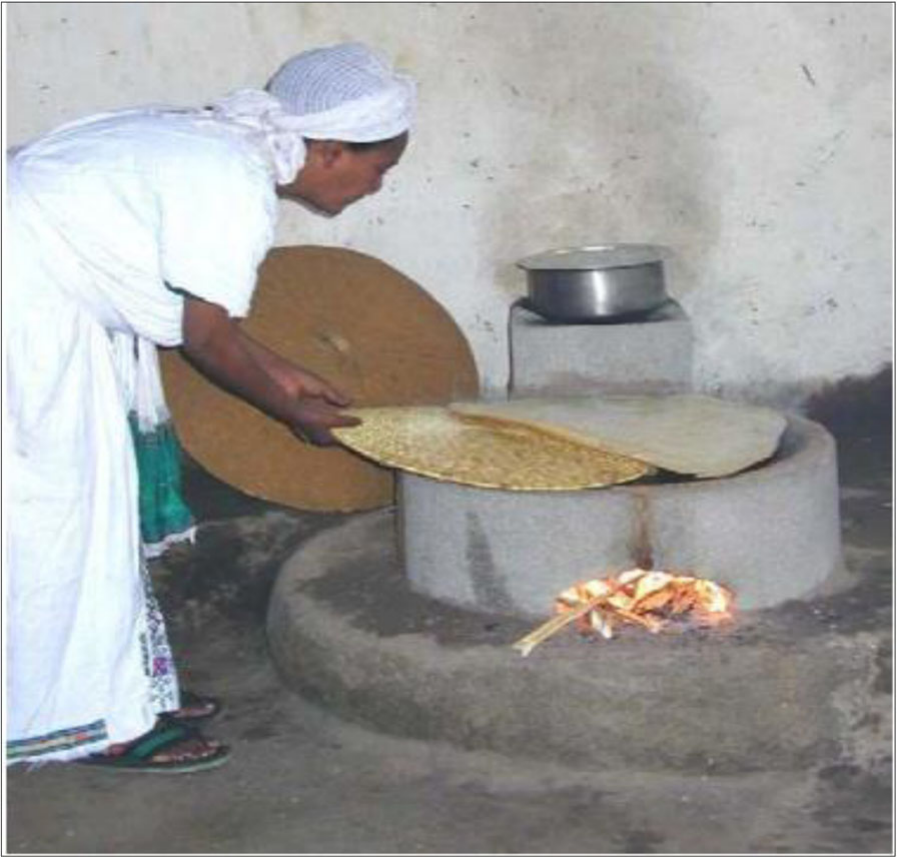
*Mirt* improved cookstove technology. Source: The World Bank [39]

In the Ethiopian context, two different household cooking devices are required traditionally. One stove is for baking the staple food of Ethiopia called *injera*, which is a unique type of yeast-risen flatbread, consumed widely in Ethiopia [38, 39], and another for other cooking purposes used nearly on a daily basis [13]. Replacing the open burning TCS method with an ICS method, locally called *Mirt* (best) improved cookstove (Fig. 1), was the intervention for this study which is the well-known commercially distributed type of ICS in Ethiopia for *injera* baking (i.e., the staple food of Ethiopia) [38, 39].

The *Mirt* is made of cement and volcanic ash and is an unvented ICS type designed by the Ethiopian Energy Studies Research to be used only for cooking *injera* in Ethiopia with a life span of at least 5 years [38, 39]. It is big in size and a fixed stove type which requires firewood to be loaded all over the cooking process [39]. It can save the quantity of fuel up to 31% compared to the TCS method [51] through efficient energy conversion in an enclosed combustion chamber which can also decrease PM_2.5_ emission up to 50% in μg/m^3^ [38, 39].

Households who were randomized to the intervention arm were identified using the permanent MHDSS house number for convenient appointment date, and the intervention was delivered at the beginning of the study period to all eligible households allocated in the intervention arm. However, since the firewood is often self-collected and inexpensive, fuel was procured by the recipients of this trial in both arms. Also, the fuel requirement was practically planned to be attained by every recipient household in the Ethiopian *Mirt* ICS implementation program.

All the trial stoves were manufactured by a local licensed firm and installed on-site by the installation teams. Demonstration in the use of the *Mirt* ICS was provided to each household during the time of installation, and the intervention was promoted regularly throughout the follow-up period by the local energy experts’ team of ICS monitors. Correspondingly, the control households were continued to use the usual open burning TCS method in an equal number of randomly allocated clusters.

Since the life span of *Mirt* ICS is about 5 years [38, 39], the length of both the intervention and the follow-up period was 1 year to safely account for seasonal factors that have a major effect on the magnitude of HAP as well as to maintain a balance between achieving sufficiently long follow-up period for HAP outcome measurement and short follow-up period to decrease attrition.

#### Trial adherence and compliance monitoring

Adherence of study households’ to the trial protocol was assessed through self-reporting and direct observation by trained field workers along with the local energy expert team. At each follow-up visit, the field workers observed and recorded the type and condition of the cookstove currently being used (i.e., no stove change or no observed breakage resulting in no use). Additionally, the primary cook was asked whether the cookstove intervention was in good working order (i.e., no reported breakage resulting in no use). Only trivial maintenance problems were detected regarding protocol adherence, and timely responses were carried out by the installation teams to avoid the possible detrimental effect of non-adherence through improving intervention protocol adherence. Besides, trial protocol compliance was checked by the local energy experts’ team of stove monitors’ through unannounced visual inspection visits in homes of both arms to enhance data validity.

#### Participant retention strategies

Once the eligible households were enrolled, a variety of strategies was used to avoid premature withdrawal of participants and the associated complexities in the analysis and interpretation of findings due to missing data. In this regard, active community engagement was established through the Ethiopian health extension program and local health development army team structure to promote participant retention and complete follow-up for the entire study period. Interest in the study was maintained through periodic communications about the intervention protocol adherence during the regular local health development army team meetings and throughout the home visits by field health workers as well as by the local energy experts.

Also, HAP measurement events were scheduled at a regular appointment of home visits to limit the participants’ burden related to follow-up visits, and at the start of the trial, control households were informed that they would receive the ICS intervention at the end of the study period to maintain justice and achieve a high level of post-recruitment participant retention.

#### Trial safety monitoring

The *Mirt* ICS intervention [38, 39], which was tested by this trial, was not involved in any drug or a medical procedure as well as not known to increase the risk of any adverse event. Nevertheless, an interim analysis was included in the protocol for safety and efficacy monitoring.

On the other hand, the *Mirt* ICS intervention [38, 39] was expected to reduce HAP-related health effects [39] and we expected participation in the intervention arm might reduce risks to the participant. Thus, any adverse events data deemed related to the trial intervention were collected and reported immediately during the routine household visits to take appropriate management as well as to inform the conduct of the ongoing and future studies. The collected data were also reviewed for safety by an independent Data Safety and Monitoring Board (DSMB) to determine whether there were grounds to stop the trial early for adverse events. Nevertheless, the Board found no grounds to stop the trial early due to adverse events.

### Household air pollution outcome assessment

Fine particulate matter with a diameter less than 2.5 μm in diameter (PM_2.5_) is a key pollutant associated with both health and climatic impacts [52], and the latest WHO indoor air quality guideline uses PM_2.5_ concentration as a key household air pollutant [20, 53]. For this reason, the status of HAP was determined by measuring the concentration of indoor PM_2.5_ using a low-cost, light-scattering particulate matter monitoring device called Dylos DC1700 air quality monitor [54, 55].

The performance of the Dylos DC1700 monitor has been previously evaluated for different scenarios in both indoor and outdoor environments [54, 56–58], and it was also found to perform well at both rural and urban locations for measuring PM_2.5_ concentration [54, 55, 59–61]. The monitor has been also utilized as a reference instrument to calibrate low-cost PM_2.5_ sensors as reported in previous studies [62], and its performance did not seem to have been impacted by aerosol composition [63], relative humidity [64], and temperature [65]. Moreover, this monitoring device is cost-effective, portable, both electric power cable and battery operated, quieter, and easier to use, and does not need laboratory facilities [54, 56, 57].

The Dylos light-scattering monitors were calibrated under actual conditions of deployment by conducting co-located PM_2.5_ concentration in μg/m^3^ measurements in randomly selected sub-sample of homes (one household per cluster) using a light-scattering monitor which allowed PM_2.5_ mass concentration measurements in μg/m^3^ following adjustment by a gravimetric method to obtain a local calibration factor as a reference to convert and correct the light-scattering (photometric) measurements.

### Data collection

At baseline, continuous indoor PM_2.5_ concentration monitoring was performed among 2031 households for one cooking hour using digital Dylos DC1700 monitors by trained environmental health officers after undergoing a 2-day HAP monitoring training. The training included how to operate the Dylos monitor, how to monitor indoor PM_2.5_ concentration using Dylos sampling protocol, how to record the acquired indoor PM_2.5_ concentration data, and how to apply standard operating procedures (SOPs). Practical exercises were carried out, and all monitors were tested as part of the training. Two senior environmental health professionals were assigned to supervise the entire HAP monitoring activity, and the overall coordination was handled by the investigators of the research project.

To measure indoor PM_2.5_ concentration, the Dylos monitors were placed in the main cooking quarter (kitchen) at least 1 m away from the edge of the stove, at a height of 1.5 m above the floor, 1.5 m away from doors, windows, and other openings horizontally [36], and at a safe location to minimize the risk of interrupting normal household activities or being disturbed. Similarly, co-located PM_2.5_ concentration measurements were also conducted for the one cooking hour in a randomly selected sub-sample of 36 households using an adjusted light-scattering monitor which allowed PM_2.5_ mass concentration measurements in μg/m^3^ of sampled air.

The HAP monitoring team members have recorded the sampling date, household ID, starting and completing time of monitoring, and the indoor PM_2.5_ concentration. The PM_2.5_ concentration data were also downloaded to a PC file by supervisors using the Dylos Logger software at the end of each monitoring day and sent to the principal investigator together with other daily records for cross-checking. Independent variables data were also collected through direct observations and face-to-face interviews using a structured questionnaire on main cooking area characteristics such as the location of the main cooking quarter, cookstove type, and frequency of the cooking event.

Subsequently, after the baseline survey and implementation of the intervention, a series of micro-environmental indoor PM_2.5_ concentration measurements were carried out by the trained environmental health officers using similar measuring devices and protocol for 1 year every 3-month interval in both arms. If houses were unavailable during the scheduled visit, repeated visits were made on any day of the same week. The duration of the follow-up period was determined to be 1 year to account for seasonal factors that might have a major effect on the magnitude of micro-environmental HAP.

### Data quality assurance

The pragmatic approach that we followed ensures the generalizability of the study findings to the wider population by maintaining the validity and reliability of findings. In this regard, a variety of measures were also taken to ensure data quality. To begin with the outcome variable, indoor PM_2.5_ concentrations were assessed in the same manner in both arms by similarly trained environmental health officers using calibrated monitors under actual conditions of deployment. Equal numbers of intervention and control households were visited every morning and afternoon in each HAP monitoring day.

The HAP monitoring team was in regular contact with the investigators of the research project with scheduled meetings, and additional communications were done as needed for feedback and quality control. To minimize the risk of bias, a specific monitoring protocol containing a detailed description of the standard operating procedures (SOPs) was used to reduce the level of error associated with PM_2.5_ concentration monitoring and other data collection through assuring consistency in measurements. Clusters were randomly allocated to intervention and control arms, all eligible households within the clusters were included in the study, allocation sequence was concealed from those assigning participant households to arms, and primary outcome assessors were blind to the intervention at baseline. A single licensed firm manufactured all the trial ICSs, and the same installation teams administered the intervention in both arms.

In addition, all initially randomized participants were analyzed in the arm they were assigned to (i.e., intention-to-treat analysis principle). Furthermore, the methodological soundness such as the large sample size that took ICC value into account, longitudinal study design, and baseline data collection on the primary outcome and related risk factors to be adjusted through Generalized Estimation Equation (GEE) modeling can help us to achieve an effective balance of potential confounders between both arms. Finally, this manuscript was reported following both the guidelines of Consolidated Standards of Reporting Trials (CONSORT) 2010 statement extension to cluster randomized trials and Template for Intervention Description and Replication (TIDieR) checklist [66] to address the essential study design components and intervention aspects of this trial report.

### Statistical analysis methods

Baseline cooking characteristics and all Dylos indoor PM_2.5_ concentration data in 0.01 ft^3^ were entered into the Statistical Package for Social Sciences (SPSS) for analysis. The Dylos DC1700 monitor measures fine particles in two size ranges. These are the small range channel, which measures 0.5-μm particulates or greater, and the large range channel, which measures particulates of 2.5 μm or greater [54, 55]. Thus, the Dylos HAP concentration data was easily calculated by subtracting the large channel value from the small channel to find the Dylos indoor PM_2.5_ concentration data in 0.01 ft^3^.

Then, linear regression analysis was performed between the Dylos HAP concentration data in 0.01 ft^3^ and the co-located monitor which allowed PM_2.5_ concentration measurements in μg/m^3^ of sampled air [60, 63]. The analysis result showed a strong linear relationship with a conversion factor of PM_2.5_ concentration in μg/m^3^ = [(6.22) (Dylos PM_2.5_ concentrations in 0.01 ft^3^) (10^−2^)]. This provides a conversion factor of 6.22, and the resulting conversion factor was used to convert the Dylos indoor PM_2.5_ concentration data in 0.01 ft^3^ to equivalent PM_2.5_ concentration data in μg/m^3^ to make the Dylos results comparable with other HAP monitors and to follow the WHO standard of measuring HAP in units of mass per volume in μg/m^3^.

To quantify the magnitude of clustering for HAP outcome at baseline, cluster-level ICC value was also calculated using a multilevel mixed-effects model (i.e., mixed-effects linear regression estimation method in STATA), which directly estimates between and within-cluster variances to calculate ICC for continuous variables. Using this method, the cluster-level ICC value for indoor PM_2.5_ concentration in μg/m^3^ was found to be 0.0346, which indicates that only 3.46% of the total variability in indoor PM_2.5_ concentration is explained by the between cluster-level variation, showing the fact that group-level characteristics are not required to explain the outcome variable. Therefore, we considered the individual households as the unit of analysis and interpretation [67] in determining the effect of ICS intervention on the longitudinal indoor PM_2.5_ concentration in μg/m^3^ compared with the continuation of the open burning TCS method.

The effect of ICS intervention on the repeated response of indoor PM_2.5_ concentration between the two arms was estimated using linear regression with GEE modeling approach among the intention-to-treat (ITT) households. The GEE analysis method is the ideal method for longitudinal data analysis due to its computational simplicity and robustness to misspecification of the repeated measures’ correlation structure. Although the GEE method is understood to be robust against a wrong choice of working correlation structure (WCS), the best WCS of the outcome variable was chosen through a critical examination of the observed correlations between subsequent measurements to get a more precise estimation of the intervention effect [68]. Using this method, an exchangeable correlation matrix was found to be most appropriate to fit the observed data. Quasi-likelihood under the independence model criterion (QIC) technique was also employed to uphold the goodness of model fitness by choosing a model with a smaller QIC value.

As a final point, our GEE analysis model has simultaneously included a continuous outcome variable of repeatedly measured indoor PM_2.5_ concentration μg/m^3^ with a binary indicator of treatment allocation (i.e., control versus intervention) as well as other indicator variables such baseline indoor PM_2.5_ concentration_,_ location of cooking quarter, secondary cookstove type used for other cooking purposes, and frequency of *injera* baking events measured at baseline.

## Results

### Participant household enrolment

**Fig. 2:**
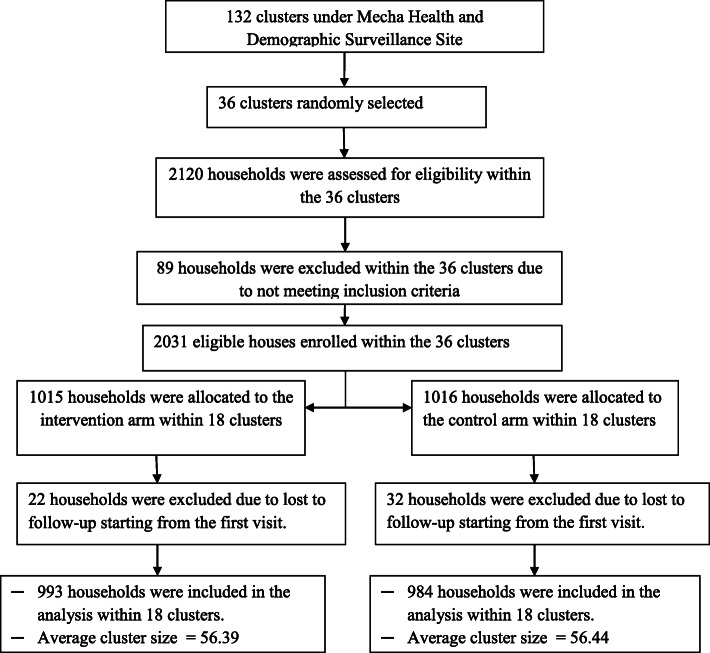
Flow diagram of the study progress from eligibility assessment to enrollment, follow-up, and analysis to test the effect of biomass-fuelled improved cookstove intervention aimed at reducing the magnitude of household air pollution compared with the continuation of an open burning TCS method

Of the 2120 houses assessed for eligibility, a total of 2031 houses fulfilled the inclusion criteria and were randomized to the intervention (*n* = 1015) or control arm (*n* = 1016) within randomly selected 18 clusters in each arm. A total of 89 (4.20%) houses were excluded at baseline due to not meeting the inclusion criteria (Fig. 2), and 54 houses (22 in intervention and 32 in the control arm) were excluded due to LTF at the first follow-up visit after initial enrollment.

### Description of household characteristics at baseline

At baseline, the mean HAP level was 855 (95% CI 839–870) as measured by indoor PM_2.5_ concentration in μg/m^3^ (850 in the control and 859 in the intervention arm), and only 3.46% of the total variability is explained by between cluster-level variation. The shared cluster-level variation for indoor PM_2.5_ concentration at baseline was also found to be comparable between arms as indicated by the estimated ICC values of 0.0317 and 0.0316 in μg/m^3^ for the control and intervention arm respectively (Table 1).

**Table 1:** Baseline characteristics of enrolled households

Characteristic		Treatment arm		Both arms (%)
		Control (%)	Intervention (%)	
Location of cooking quarter	Inside living house	415 (40.8)	327 (32.2)	742 (36.5)
	Separate kitchen	601 (59.2)	688 (67.8)	1289 (63.5)
	Total	1016 (100)	1015 (100)	2031 (100)
Secondary cookstove type	Traditional	936 (92.1)	924 (91.0)	1860 (91.6)
	Improved	80 (7.9)	91 (9.0)	171 (8.4)
	Total	1016 (100)	1015 (100)	2031 (100)
Frequency of *injera* baking event	Everyday	131 (12.9)	134 (13.2)	265 (13.0)
	Every other or more days	885 (87.1)	881 (86.8)	1766 (87.0)
	Total	1016 (100)	1015 (100)	2031 (100)
PM_2.5_ concentration in μg/m^3 a^		850 (358.6)	859 (359.3)	855 (359)
Cluster-level ICC for PM_2.5_ concentration in μg/m^3^		0.0317	0.0316	0.0346

^a^Standard deviations are shown in parentheses.

### Household air pollution follow-up

The first follow-up visit was carried out in the spring season of the year 2018 (October to November), the second round took place in the summertime of the year 2019 (January to February) which is the dry season of the year in Ethiopia, the third occurred in fall/autumn season of the year 2019 (April to May), and the fourth round of the HAP monitoring round occurred in the winter (rainy) season of the year 2019 (July to August). This trial study was terminated at the planned target of 1 year after the last participant household had been randomized.

Follow-up data were obtained from 1977 households (984 in the control and 993 in the intervention arm) at least for one indoor PM_2.5_ concentration measurement which established the ITT population dataset within 18 clusters in each arm which were included in each analysis with an average cluster size of 54.92 with a standard deviation of 9.75 for the ITT population. Also, since the life span of *Mirt* ICS is about 5 years [38, 39], only trivial maintenance problems were detected regarding protocol adherence, and timely responses were carried out by the installation teams to avoid the possible detrimental effect of non-adherence. Nevertheless, 327 houses (166 in intervention and 161 in the control arm) were LTF during the entire follow-up period which gave a total of 718 LTF observations in both arms which were automatically excluded in each analysis (Additional file 1). Among which 8.2%, 19.1%, 31.5%, and 41.2% of the LTF observations occurred in the first, second, third, and fourth rounds, respectively.

### Household air pollution outcome estimations

The post-intervention, hourly longitudinal mean indoor PM_2.5_ concentration was estimated to be 635 (95% CI 627–642) in μg/m^3^ (465 in the intervention and 805 in the control arm) among the ITT households. It showed an overall reduction of about 46% from the baseline value of 859 (95% CI 837–881) to 465 (95% CI 458–472) in the intervention arm compared to only about 5% reduction from 850 (95% CI 828–872) to 805 (95% CI 794–817) in the control arm. This represents about a 58% reduction in mean longitudinal indoor PM_2.5_ concentration in μg/m^3^ in the intervention arm compared to the control arm.

**Fig. 3:**
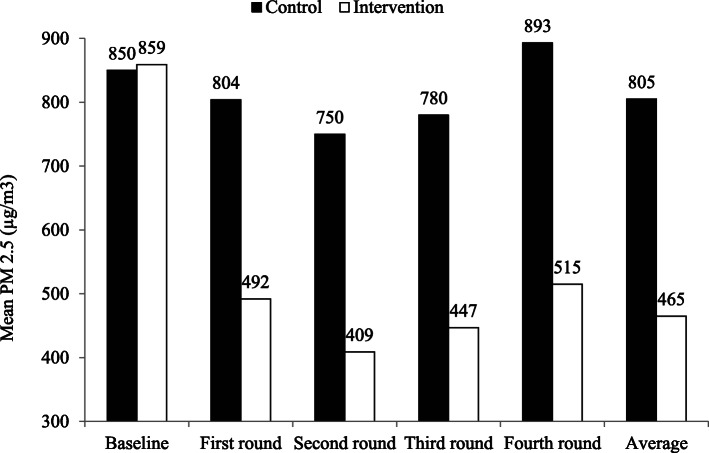
Mean indoor PM_2.5_ concentration in μg/m^3^ by round and arm

The overall mean indoor PM_2.5_ concentration showed a variation by round in a similar fashion in both arms. The distribution for the mean indoor PM_2.5_ concentration by round was found to be 648 (95% CI 633–662), 579 (95% CI 564–594), 613 (95% CI 598–629), and 703 (95% CI 686–720) for the first, second, third, and fourth rounds, respectively, as depicted next in the treatment arm in Fig. 3.

### Estimation of intervention effect

Regarding the intervention effect, the use of the current biomass-fuelled ICS intervention significantly reduced the longitudinal mean indoor PM_2.5_ concentration by about 343 μg/m^3^ with an estimated beta coefficient (*Ḃ*) of − 343 (95% CI − 350, − 336) compared with the continuation of an open burning TCS, while other independent variables are held constant. The longitudinal change in mean indoor PM_2.5_ concentration was significantly associated with baseline mean indoor PM_2.5_ concentration with an estimated beta coefficient (*Ḃ*) of 0.765 μg/m^3^ (95% CI 0.750, 0.781) which demonstrates an increase by 0.765 μg/m^3^ for every unit increase in the baseline mean indoor PM_2.5_ concentration. The findings of this study also showed that the longitudinal change in mean indoor PM_2.5_ concentration was significantly associated with stove type used for other cooking purposes and frequency of the *injera* baking event measured at baseline as demonstrated next by the GEE analysis model in Table 2.

**Table 2:** Effect of the improved cookstove intervention on the concentration of household air pollution in micrograms per cubic meter of sampled air

Parameters		B	S.E.	95% Wald C.I.		*P* value
				Lower	Upper	
Treatment arm	Intervention	− 342.992	3.7307	− 350.304	− 335.680	.0001*
	Control	1				
Baseline indoor PM_2.5_ concentration in μg/m^3^		0.765	0.0078	0.750	0.781	.0001*
Location of main cooking quarter	Inside living house	1				
	Separate kitchen	− 6.994	4.0553	− 14.942	0.954	.085
Secondary cookstove type	Traditional	1				
	Improved	− 16.976	7.6349	− 31.941	− 2.012	.026*
Frequency of *injera* baking event	Everyday	1				
	Every other or more days	− 42.656	6.7964	− 55.976	− 29.335	.0001*

*Significantly associated at *p* value < 0.05

## Discussion

The magnitude of the baseline HAP observed in this study (855 μg/m^3^ (95% CI 839–870)) is nearly comparable to those reported in previous studies in Ethiopia [21, 22] but more than 34 times higher than WHO guideline values [20]. The possible explanation for the higher HAP in the current study might be the reflection of the complete biomass fuel use as a primary household energy source by all households, presence of extra indoor burning events such as coffee ceremony, burning incense, and local alcohol/*areqi* making as well as the lower ICS adoption level as indicated in our earlier research work carried out in the current study area [13, 19]. The other possible explanation might be the reflection of the difference in HAP monitoring duration.

The WHO guideline values are developed based on 24-h period HAP measurement [20]. In our case, given the important role of peak emission periods in determining the total daily HAP exposure and the concentration of PM_2.5_ is higher during the smoldering period in rural kitchens [69], the concentration of HAP was measured for the 1-h duration in the smoldering period to consider the “worst-case scenario” in the concentration of HAP, which gives a better understanding about the determinants of HAP concentration. This, also, avoids many of the complexities surrounding the 24-h period HAP measurement in a rural setting.

The post-intervention, overall mean indoor PM_2.5_ concentration was found to be 635 (95% CI 627–642) in μg/m^3^ (465 in the intervention and 805 in the control arm). It showed a variation by round in a similar fashion in both arms with a steep reduction followed by a slight increase in a parallel manner in both arms. A maximum concentration of 703 μg/m^3^ (95% CI 686–720) was observed at the fourth round which was carried out in the winter (rainy) season in Ethiopia. Thus, the observed highest mean indoor PM_2.5_ concentration at the fourth round could be due to the combined effect of longer burn times, lower ventilation rates, and other behavioral factors that might lead to an increase in the magnitude of HAP in winter season as evidenced by a previous study [70].

Concerning intervention effect, the longitudinal assessment of HAP concentration showed that use of the current biomass-fuelled ICS intervention significantly reduces the indoor PM_2.5_ concentration by about 343 μg/m^3^ with an estimated beta coefficient (*Ḃ*) of − 343 (95% CI − 350, − 336; *p* < 0.001) compared with the continuation of an open burning TCS. We interpret the current finding as empirical evidence for the presence of the intervention effect. The overall reduction was found to be about 46% from the baseline value of 859 (95% CI 837–881) to 465 (95% CI 458–472) in the intervention arm compared to only about 5% reduction from 850 (95% CI 828–872) to 805 (95% CI 794–817) in the control arm. This represents about a 58% reduction in mean indoor PM_2.5_ concentration in the intervention arm compared to the control arm.

Our result is almost comparable to the previous randomized controlled ICS trials which reported significant reductions for micro-environmental HAP concentrations to 485 (46%), 320 (52%), and 119 (59 %) in μg/m^3^ in Rwanda [30], Ghana [31], and India [26], respectively, following biomass-fuelled ICS interventions. The current finding is also broadly comparable to a recent multicounty randomized controlled ICS trial that reported significant reductions for mean PM_2.5_ personal exposure in μg/m^3^ to 95.1 (31%), 31.1 (32%), and 32.4 (65%) in Uganda, Vietnam, and Kyrgyzstan, respectively, following local biomass-fuelled ICS interventions [71]. Also, six ICS interventions aimed at reducing HAP in rural Kenya have achieved 18 to 45% reductions in mean kitchen PM_2.5_ levels [72].

On the other hand, our result is in contrast with that of a recent trial in Rwanda [33]. The Rwanda large-scale stove trial reported that a biomass-fuelled ICS intervention had no significant impact on personal exposure to PM_2.5_ concentration among primary cooks (*Ḃ* = − 0.089, *p* = 0.486) and children (*Ḃ* = − 0.228, *p* = 0.127). The possible explanation for the difference in findings might be linked with the difference in HAP monitoring methods in which HAP concentration reduction in personal monitoring method tends to be lower than reductions in the micro-environmental HAP concentration monitoring. Further research is perhaps needed using a personal exposure monitoring method directly to establish whether the use of the current biomass-fuelled stove could be translated into meaningful HAP reduction as well as into health benefits.

In the present study, the other finding worth highlighting is related to the type of cookstove used for extra cooking purposes at baseline. We observed a significant reduction in the longitudinal indoor PM_2.5_ concentration associated with the use of improved stove for extra cooking purposes with an estimated beta coefficient (*Ḃ*) of − 17 (95% CI − 32, − 2; *p* < 0.001) compared with the use of TCS method. Similar results were observed by previous cross-sectional studies conducted in Ethiopia which reported a reduction of PM_2.5_ concentrations associated with improved stove use compared to the traditional type of stove [21, 22]. Thus, the use of the traditional stove for extra cooking has appeared as one contributor to the longitudinal indoor PM_2.5_ concentration, and the use of an ICS may reduce HAP in the study area.

In addition, the longitudinal change in mean indoor PM_2.5_ concentration was significantly associated with the frequency of *injera* baking event at baseline with estimated beta coefficients (*Ḃ*) of − 43 (95% CI − 56, − 29; *p* < 0.001) which implies that those households who bake every other or more days significantly reduce the longitudinal mean indoor PM_2.5_ concentration by about 43 μg/m^3^ compared with everyday baking event frequency. This might be due to high HAP emissions from several periods of intense cooking which might determine HAP as evidenced by the previous study conducted in Ethiopia. At last, the most important implication of the major finding of this trial is that the biomass-fuelled ICS intervention significantly reduces the concentration of household air pollution compared to the TCS method.

Concerning health benefits, unvented biomass-fuelled ICS solutions are likely to have no or minimal impact on serious HAP-linked health conditions [73], and vented (i.e., with chimney) rocket biomass-fuelled ICSs can likely have small but meaningful health benefits [74]. Only well-performing fan gasifiers and natural draft gasifier biomass-fuelled ICS interventions maintain the potential to significantly reduce the incidence of HAP-linked serious illnesses [75], and switching from biomass-fuelled cookstove technologies to cleaner fuel stoves, such as electricity, LPG, biogas, ethanol, or solar cooking, is likely to bring about the required health impact through the largest reductions in HAP [5].

These biomass-fuelled basic ICS technologies are not, however, completely without health benefit, because improvements in minor health problems, such as eye irritation, headache, and respiratory discomfort, are widely reported following basic ICS interventions [76]. Also, the basic ICS interventions would achieve economic and environmental benefits by reducing household fuel consumption as well as by the direct reduction of the amount of wood burned which can, in turn, decrease environmental destruction and pollution [77].

As to generalizability of findings, this fairly large-sample cookstove trial was completed effectively from a methodological and practical point of view, and our sample was comparable to the wider population of households that use biomass-fuelled cookstoves as a major household energy source for cooking purposes throughout Ethiopia. Also, this trial study was done in one of the LMICs where most households use biomass fuels for cooking [12, 53]. Hence, any effect of the biomass-fuelled ICS intervention on the concentration of HAP found in this trial study should be generalizable to other households with high levels of HAP in Ethiopia, and other similar settings in LMICs

## Limitations

Perhaps, this is the first large-scale longitudinal biomass-fuelled ICS trial that monitored the concentration of HAP under field conditions in Ethiopia. There were, however, certain important limitations to our trial. Specifically, we acknowledge the unblinded nature of the cookstove intervention, and the lower limit of fine particulate matter fraction that the Dylos DC1700 device can detect is limited to 0.5 μm only. This might affect the concentrations of HAP measured at both baseline and follow-up and would probably decrease our ability to detect differences between the groups. Nevertheless, the margin for such effect should be small as there was no difference in the use of the Dylos DC1700 HAP measurement between the intervention and control arms. Also, budget limitations did not permit personal PM_2.5_ assessment, a more reliable metric for HAP associated with health outcomes [78], and although we tried to collect objective indicators of stove use by undertaking visual observations, the study relied heavily on reported data, which is susceptible to reporting bias.

## Conclusions

Based on the findings of this longitudinal assessment, the biomass-fuelled ICS intervention significantly reduces the concentration of household air pollution compared to the TCS method. This suggests that the implementation of these cookstove technologies may be necessary to achieve household air pollution exposure reductions.

## Supplementary Information

**Additional file 1.** Tabular presentation of lost to follow-up events during the entire follow-up period for the trial study entitled biomass-fuelled improved cookstove intervention to prevent household air pollution in Northwest Ethiopia. Note: Among the total lost to follow-up of 718*, only 327** houses (161 in the control,166 in the intervention arm) were recorded as new incidences during the entire follow-up period, and 391 were registered as repeated lost to follow-up observations in both arms.

## Data Availability

The data that support the findings of this study is available from the corresponding author on reasonable request and with permission of the “Mecha” Health and Demographic Surveillance research center at Bahir Dar University in Ethiopia.

## Abbreviations

Β: Beta coefficient
CI: Confidence interval
GEE: Generalized Estimation Equation
HAP: Household air pollution
ICC: Intra-cluster correlation coefficient
ICS: Improved cookstove
ITT: Intention-to-treat
LMICs: Low- and middle-income countries
LTF: Lost to follow-up
MHDSS: Mecha Health and Demographic Surveillance System
PM_2.5_: Particulate matter less than 2.5 micrometers in diameter
TCS: Traditional cookstove
WCS: Working correlation structure
WHO: World Health Organization
