# Supplementary material for: Biomass-fuelled improved cookstove intervention to prevent household air pollution in Northwest Ethiopia: a cluster randomized controlled trial
Source: Environ Health Prev Med. 2021 Jan 4;26:1. doi: 10.1186/s12199-020-00923-z (PMC7783973; doi:10.1186/s12199-020-00923-z)
Supplement: Supplementary file 1 — Additional file 1. Tabular presentation of lost to follow-up events during the entire follow-up period for the trial study entitled biomass-fuelled improved cookstove intervention to prevent household air pollution in Northwest Ethiopia. Note: Among the total lost to follow-up of 718*, only 327** houses (161 in the control,166 in the intervention arm) were recorded as new incidences during the entire follow-up period, and 391 were registered as repeated lost to follow-up observations in both arms. [file 12199_2020_923_MOESM1_ESM.pdf]

Characteristics		Treatment arm		Total (%)	
		Control (%)	Intervention (%)		
LTF	Yes	373 (9.2)	345 (8.5)	718* (8.8)	X^2 = 0.280 (*P=0.291*)
	No	3691 (90.8)	3715 (91.5)	7406 (91.2)	
	Total	4064 (100)	4060 (100)	8124 (100)	
Reasons for lost to follow-up	Moved out of study area	83 (51.55)	91 (54.82)	174 (53.21)	
	Consent withdrawal	62 (38.51)	44 (26.51)	106 (32.42)	
	Not at home	16 (9.94)	31 (18.67)	47 (14.37)	
	Total	161 (100)	166 (100)	327 (100)**	
